# Neural adaptation to non-symbolic number and visual shape: An electrophysiological study

**DOI:** 10.1016/j.biopsycho.2014.09.006

**Published:** 2014-12

**Authors:** Fruzsina Soltész, Dénes Szűcs

**Affiliations:** Centre for Neuroscience in Education, Department of Psychology; University of Cambridge, Cambridge, UK

**Keywords:** EEG, Numerical cognition, Neural adaptation, Number sense, Number comparison

## Abstract

Several studies assumed that the analysis of numerical information happens in a fast and automatic manner in the human brain. Utilizing the high temporal resolution of electroencephalography (EEG) in a passive oddball adaptation paradigm, we compared event-related brain potentials (ERPs) evoked by unattended shape changes and unattended numerosity changes. We controlled visual stimulus properties in a stringent manner. Unattended changes in shape elicited significant, gradual adaptation effects in the range of early visual components, indicating the fast and automatic processing of shapes. Changes in numerosity did not elicit significant changes in these early ERP components. The lack of early number-specific effects was qualified by a significant interaction between Shape and Number conditions. Number change elicited gradual ERP effects only on late ERP components. We conclude that numerosity is a higher-level property assembled from naturally correlating perceptual cues and hence, it is identified later in the cognitive processing stream.

## Highlights

• We compare EEG indicators of automatic change detection to numbers vs shape.
• We apply stringent control of perceptual variables.
• Shape change is detected early in the cognitive processing stream.
• Number change is detected only later in the cognitive processing stream.
• Nonsymbolic number is not a perceptual property extracted automatically.

## 1 Introduction

Several neuroimaging studies have investigated the nature of a putative abstract, evolutionarily grounded approximate magnitude representation which may be hosted in the intraparietal sulcus (IPS) (see Dehaene, Molko, Cohen, & Wilson, 2004). The most widely used markers of this magnitude representation are numerical distance and ratio effects (Moyer & Landauer, 1967). These refer to the phenomenon that it is more difficult to discriminate closer (ratio closer to 1, e.g. 8:7) than further away (ratio further from 1; e.g. 1:2) numerosities. Several recent studies used non-symbolic magnitude adaptation tasks (participants adapt to the number of items in dot displays) to detect brain correlates of the magnitude representation (see Ansari, 2008 for review). However, both earlier and recent research made it clear that non-symbolic number discrimination tasks are seriously confounded with visual stimulus cues (Fuhs and McNeil, 2013, Gebuis and Reynvoet, 2011, Gebuis and Reynvoet, 2012a, Gebuis and Reynvoet, 2012b, Gebuis and van der Smagt, 2011, Gilmore et al., 2013, Mix et al., 2002, Mix et al., 1997, Szűcs et al., 2013). Therefore, results have to be interpreted with caution and further research is necessary to determine exactly how visual stimulus parameters and numerical parameters are evaluated in non-symbolic magnitude discrimination tasks.

Numerous studies have used symbolic and non-symbolic number discrimination tasks. In symbolic tasks typically two numbers are shown (e.g. 3 vs 4) and participants decide which one is larger. In non-symbolic magnitude discrimination tasks participants typically see two dot patterns on a screen and decide which one is more numerous. Numerical distance and ratio effects have been demonstrated in various functional magnetic resonance imaging studies (fMRI; e.g. Ansari et al., 2006, Cohen-Kadosh et al., 2007, Kawashima et al., 2004, Notebaert et al., 2010, Pinel et al., 2004). Similar effects have been demonstrated by electro-encephalography studies (EEG; Grune et al., 1993, Soltész et al., 2007, Szűcs and Csépe, 2004, Szűcs and Csépe, 2005; Szűcs et al., 2007). The majority of these studies used symbolic numbers and/or had explicit number-related instructions (e.g. asking for explicit number comparison judgments), bringing the concept of abstract numbers to the participants’ attention. Hence, several previous studies may have measured the brain correlates of general comparison activity rather than effects related to number representations (Fias et al., 2013, Van Opstal and Verguts, 2011). In order to avoid this problem a handful of studies used neural number adaptation paradigms where no explicit number related response was required and it was assumed that participants may not even be conscious of implicit number processing requirements.

Adaptation is the phenomenon when the level of response, either behavioural or neural, decreases when a certain type of stimulus is being repeated, even when the experimental aspects of the stimulus are disguised (also called “neuronal savings”, “repetition suppression”, etc.; for reviews see Grill-Spector et al., 2006, Krekelberg et al., 2006). The adaptation paradigm typically consists of a stream of *standard* stimuli and occasional *deviant* stimuli. During the adaptation stream, a certain property (i.e. numerosity) of the stimuli is being kept constant. After the adaptation stream, a deviant item is shown to which there is a *rebound* in the response (i.e. increased looking time or increased neuronal activity) if the change in the given stimulus property is registered by the cognitive system. Since number adaptation paradigms involve either passive viewing, or a non-related distracter task (for example participants are asked to detect a colour change in the fixation cross which is presented independently of the stimulus stream used to induce adaptation), it can be assumed that neither task difficulty, nor response selection or attention would interfere with number-related cognitive processes. Although the neural mechanism behind adaptation is not yet fully understood, adaptation is now a widely used technique in several fields of cognition. Neuronal adaptation has been shown at the level of sensory features, at the level of somewhat more abstract perceptual properties, and also at the level of categorical–conceptual properties, like face, word meaning or numerosity (for review, see Grill-Spector et al., 2006, Piazza et al., 2007).

Brain imaging number adaptation studies usually test for the parametric modulation of brain activity, evoked by the parametric manipulation of numerical distance/ratio between standard and deviant stimuli. The parametric modulation is measured in terms of the amount of rebound evoked by a deviant stimulus in comparison to the activity in response to the preceding standard stimuli. Most fMRI studies reported parametric modulations in function of number in the IPS (Ansari et al., 2006, Cohen-Kadosh et al., 2007, Hsu and Szűcs, 2012, Notebaert et al., 2010, Piazza et al., 2007, Pinel et al., 2004). Although excellent localization of active brain areas is possible with fMRI, the methods drawback is that its time resolution is relatively poor, especially when compared to the time resolution of EEG. As a consequence, several cognitive events and processes may overlap and thus contribute to the observed effects in an fMRI measurement. Meanwhile, EEG provides a time resolution at the millisecond level and has already been used to disentangle functionally separate cognitive processes which occur in rapid succession.

Early and late cognitive events have been identified during numerical processing (Dehaene, 1996, Hyde and Spelke, 2012, Libertus et al., 2007, Pinel et al., 2001, Soltész et al., 2007, Soltész et al., 2011, Szűcs and Csépe, 2004, Szűcs and Csépe, 2005, Szűcs and Soltész, 2008; Szűcs et al., 2007; Temple & Posner, 1998). The numerical distance effect already modulated ERP amplitude at around 200 ms after stimulus presentation, indicating a fast and automatic processing of numerical magnitudes. This supposedly number-specific ERP component emerging over the parietal areas around 200 ms after stimulus presentation has been termed the P2p (Dehaene, 1996; symbolic stimuli). Following the early effect of numerical distance, modulations of ERP amplitude have been found at later time intervals as well. These ERP components are regarded as indices of domain-general processes and are related to categorical decisions (P300; Donchin, 1981) or to explicit recognition memory (P600; Friedman & Johnson, 2000). Utilizing the non-symbolic number adaptation paradigm in an EEG experiment, Hyde and Spelke (2012) replicated and extended earlier findings on the P2p ERP component (Dehaene, 1996, Libertus et al., 2007, Temple and Posner, 1998). The amplitude of P2p was claimed to be sensitive to numerical manipulations (distance effect) and was localized mainly to the right intraparietal regions. The P2p response is larger when the current number (magnitude) is closer to, hence less discriminable from, the previous magnitude (Hyde & Spelke, 2012). The authors concluded that the P2p is an index of the approximate magnitude representation (Hyde & Spelke, 2012).

Most recent studies have used non-symbolic magnitude adaptation tasks which are generally considered to be more appropriate measures of the evolutionarily primitive magnitude representation than symbolic tasks. However, a major problem with non-symbolic magnitude tasks is that it is impossible to control for visual stimulus confounds co-varying with number in each individual trial. Hence, adaptation effects can just as well rely on numerical adaptation as on adaptation to visual confounds co-varying with number. For example, in active comparison tasks such visual confounds can have a profound effect on performance even when attempts are made to control for visual parameters across the whole experiment (Fuhs and McNeil, 2013, Gebuis and Reynvoet, 2011, Gebuis and Reynvoet, 2012a, Gebuis and Reynvoet, 2012b, Gebuis and van der Smagt, 2011, Gilmore et al., 2013, Szűcs et al., 2013). Practically all fMRI and EEG non-symbolic adaptation studies are exposed to this stimulus confound problem. For example, in the study of Piazza, Izard, Pinel, Le Bihan, and Dehaene (2004) the surface and density of the dot displays were varied independently of number, the circumference indeed still correlated with changes in numerosity (for the control of perceptual correlates in detail see Gebuis & Reynvoet, 2011). Hence, if surface is being controlled while changing the numerosity, circumference still changes in function of numerosity. Second, in a follow-up paper (Piazza et al., 2007) it has been noted that the numerical aspect of the experiment was made explicit to participants. As soon as the “purpose” of the study is brought to the attention of participants, conscious strategies and/or simple, conscious change detection can no longer be disentangled from adaptation. In summary, the fMRI adaptation paradigms contain either *perceptual confounds* (e.g. circumference; Cantlon et al., 2006, Piazza et al., 2004) or *confounds from task instruction* (Piazza et al., 2007), both probably leading to a conscious *detection of change* instead of adaptation, undermining conclusions on automatic number processing. The non-symbolic adaptation paradigm used in the aforementioned EEG study (Hyde & Spelke, 2012) also contains a perceptual confound which overlaps with numerical changes, leaving it difficult to separate numerical and perceptual processes from each other. In this paradigm (Xu & Spelke, 2000), sum surface and density of the displays in each trial were varied in such a way that ideally nothing but the number changed from habituation to test trials. However, the distribution of item sizes across trials was very different from the distribution of the summary surface across trials and it was correlated with the numerosity of the dots (for a discussion of parameter details see Soltész, Szűcs, & Szűcs, 2010). Again, due to confounds, the results might reflect a *sensory change detection process*, rather than *adaptation specifically to number*. Recent studies have also explicitly demonstrated that participants strongly rely on visual cues (even when they are carefully controlled) when judging the relative numerosity of sets (Gebuis and Reynvoet, 2011, Gebuis and Reynvoet, 2012a, Gebuis and Reynvoet, 2012b, Gebuis and van der Smagt, 2011).

In order to be able to tell *number-specific processes* apart from *sensory change detection*, a paradigm comparing responses to change in numerosity to responses to change in perceptual features is required. For this purpose, we have substantially modified the number adaptation paradigm previously used in fMRI studies (Piazza et al., 2004). We manipulated both the surface and the circumference of stimuli at the level of individual items and that of the whole display, so that for half of the stimuli surface parameters, and for the other half circumference parameters were kept constant, preventing a reliable correlate of numerosity across all stimuli. We contrasted neural responses elicited within a block where number was manipulated to neural responses elicited within a block where item shapes were manipulated in a similarly parametric manner to see whether number-specific processes arose early, in an automatic manner. We expected that if the cognitive representation of numbers was domain-specific and accessed in an automatic manner, such as that of other visual features like shape, we should expect the modulation of early ERP components (i.e. the P2p component), reflecting automatic and feedforward processes (Henson, Mouchlianitis, Matthews, & Kouider, 2008), by numerical distance in the Number condition. If the early and automatic recognition of abstract numerosity is due to perceptual change detection, as we hypothesize, the supposedly number-specific P2p should be absent after changes in numerosity since there is no consistent perceptual confound across the trials in this paradigm. To avoid attentional confounds, a distractor task was applied in both conditions.

## 2 Methods

### 2.1 Participants

Participants were recruited through advertisement at the University of Cambridge, U.K. Volunteers were reimbursed for their participation. In total, 19 participants volunteered to participate in the study (Mean age: 25.21; range: 21–32; 11 females). All volunteers had normal or corrected to normal vision. Two participants’ data have been discarded due to large noise in the EEG data (for further details please see Section 2.4 Two subjects, with less than 30% trials left were excluded from further analysis.). All the volunteers were students of the Faculty of Education, University of Cambridge, U.K., aged between 21 and 28 years old. The study was approved by the Psychology Research Ethics Committee of the University of Cambridge.

### 2.2 Stimuli

**Fig. 1:**
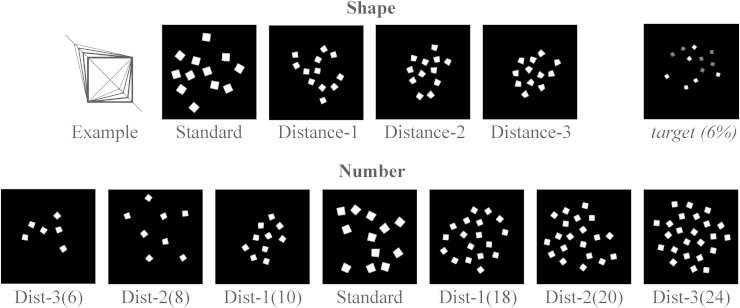
Illustration of stimuli for the shape and number conditions. For shape, the panel called ‘Example’ shows the magnified version of shapes as distortions of a square along the diameter.

Fig. 1 illustrates the stimuli, which consisted of sets of rectangular white shapes presented on a black background. The stimuli were presented for 250 ms with an ISI of 900 ms. Two oddball conditions were applied to test adaptation effects separately for Number and Shape.

In the *Shape condition*, the number of items per one display was randomly varied, with all the different numbers (6, 8, 10, 12, 18, 20, 24; numbers also used in the Number condition, see below) appearing at equal times. The shapes of the items were systematically varied: six sevenths of the stimuli were standard and one seventh of the stimuli were deviants. It total, there were 1176 standard shape displays and 168 deviant shape displays. The deviants were divided into three different types, or distances, depending on their similarity to the standard shape. The standard shape was a square. The deviants were transformed from the square, in the following way. In ‘distance 1’, one of the two diagonals of the square was shifted by 1/16 which resulted in a quadrangle with one narrow and three wider angles. In ‘distance 2’, the diagonal was shifted by 1/8. In ‘distance 3’, the diagonal was shifted by 3/16 (see Fig. 1). In the *Number condition*, all shapes were presented at equal times, in a random order. Meanwhile, the number of items on a display was varied in as follows. It total, there were 1176 standard number displays and 168 deviant (6/7 of total) number displays. The standard display consisted of twelve items. For deviants, numbers smaller and larger than 12 were used, in order to avoid simple size effects. Using both smaller and larger deviants prevent that the possible numerical distance effects would purely be due to a reaction to ‘increase’ or ‘decrease’, instead of numerical distance. Our paradigm also conforms with previous number adaptation experiments, where both smaller and larger deviants have been used (e.g. Pinel et al., 2004). Distance 1 were numbers 10 and 18, distance 2 were 8 and 20 and distance 3 were 6 and 24 (approximately 3/4, 2/3, and 1/2. It is approximate because in some cases the exact number would have been a decimal number so rounding was necessary). Presentation order of the Shape and the Number blocks were counterbalanced across subjects. Each block consisted of two sets (approx. 8 min each) with a short break in between sets.

As surface and circumference are dependent functions, they cannot be manipulated independent of each other. If for example overall surface is kept constant across different numerosities, then overall circumference will perfectly correlate with the number of items, and vice versa. For example, if overall surface (the summary of all items surface) is kept the same across numbers 12 and 24, then overall circumference (the summary of all items circumference) will correlate with the number of items,^1^ providing a systematic clue of numerosity across all the trials. One way to decrease this unavoidable confound is to intermix trials controlling for circumference and surface. Furthermore, both the extensive (summary of the items’ surface or circumference) and the intensive (each item's surface or circumference) properties were controlled for. Taking both item- and summary parameters into account is important, because for example when overall surface is being kept constant across numerosities, then as numerosity increases, each item in the display decreases, providing a possible constant correlate of numbers. It is also important to note that it is impossible to control each of these physical parameters at the same time. However, by varying more of them, the possibility that volunteers recognize and ‘follow’ a constant confounding cue of numerosity across trials decreases. In other words, in some trials it is item surface that correlates, in other trials it is overall circumference, but since they are randomly intermixed the confounds will not be constant and will less likely induce numerical distance-like effects. We have generated standard and deviant stimuli via equating numerosities along overall surface, or overall circumference, or item surface, or item circumference, at equal times (1/4th of the trials). If either of the parameters would have caused systematic artefacts, we might have found significant effects in the ERPs. There were no significant effects in the Number condition, which could have been attributed to any systematic artefacts.

However, it is important to note that in the *Shape condition* either surface or circumference changes indicate the change of the shape. Although both surface and circumference were equated on the grand average across the experiment both as extensive and as intensive properties, the trial-by-trial correlation is unavoidable. The ratio of surface and circumference is different for each shape, which might provide a perceptual cue different from shape in the *Shape condition.* This possible perceptual confound was completely removed from the *Number condition* – shapes randomly varied within each numerosity.

The items were arranged on the display based on a random grid generated in Matlab (MathWorks). The grid never contained canonical arrangements (e.g. shapes of the dice), changed from display to display and was controlled for size so that edges of the items could never overlap. The density of the grid was also randomly varied, so that density did not correlate with numerosity. In order to make size and number changes appear even more random, the size of, for example the 12-item-display item (standard), was also randomly varied across displays (within 5%).

The target stimulus was a display of items, half of which were grey instead of white (∼6% of the trials). Target stimuli provided a cover task (i.e. not number- or shape specific instructions) for the experiment.

### 2.3 Task

Participants were asked to depress a button with their right thumb when a target stimulus appeared. The numerosity of items or numbers in general was never mentioned to the participants (contrary to Piazza et al., 2007) in order to avoid the possibility that they direct their overt attention to the numerical magnitude property of the displays. Furthermore, during a short debriefing after the experiment, volunteers were asked whether they noticed throughout the experiment a change in: (1) the number; (2) the shape and; (3) the size of the items in the display. Also, if such changes were noted, they were asked whether; (4) they thought that these changes were systematic in some way.

### 2.4 EEG recording and pre-processing

EEG data were recorded in a Faraday chamber and digitized with a 24-bit A/D converter using the 129-channel EGI Geodesic Sensor Net system. The sampling rate was 500 Hz. Data pre-processing was done using Matlab. The data were highpass filtered at 0.01 Hz and lowpass filtered at 50 Hz offline, using a two-directional (non-phase shift) second order Butterworth filter. Epochs from −200 to 800 ms (stimulus at 0 ms) were extracted and baseline-corrected to the −200 to 0 ms period. Data were re-referenced from electrode Cz to the linked mastoids. Epochs containing data points over or below ±100 μV, or with large eye-movements as determined by inspection, were marked for rejection. Electrodes showing stationary and non-movement related noise across the experiment were interpolated (maximum 5 electrodes in one participants data). Sixty-four percent of trials were kept for analysis (65% in the Number condition and 63% in the Shape condition. Range: 51.2–93.7%). Two subjects, with less than 30% trials left were excluded from further analysis.

### 2.5 ERP – interval analysis

Event-related potentials were calculated for the standard and for the three distance conditions (d1–d3) within each oddball condition (Shape, Number).

**Fig. 2:**
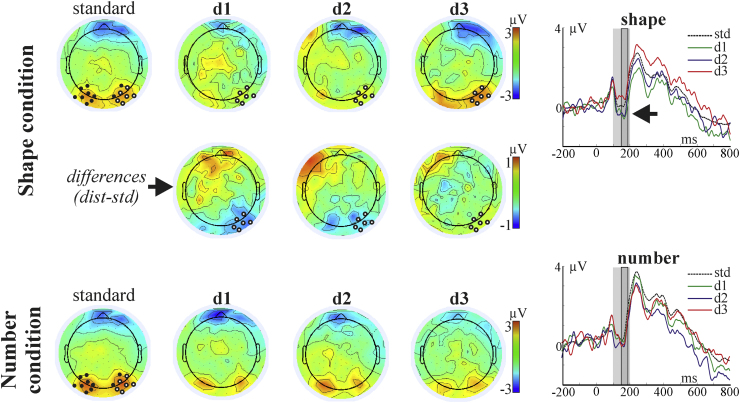
Left: Topographic plots of the N1 component from the significant time interval (150–190 ms) for the standard and for the three distance conditions. Difference (distance minus standard) topographic plots are presented for the Shape condition. The 16 electrodes entered into the analysis are marked on the standard plot. Electrodes which showed the significant distance effect (after FDR correction, see main text) are marked on the distance plots. Right: Time domain ERP, averaged from the electrodes showing the significant distance effect in the Shape condition. The significant (FDR corrected) time interval is marked with grey. The time interval initially entering the analysis is marked by light grey.

**Fig. 3:**
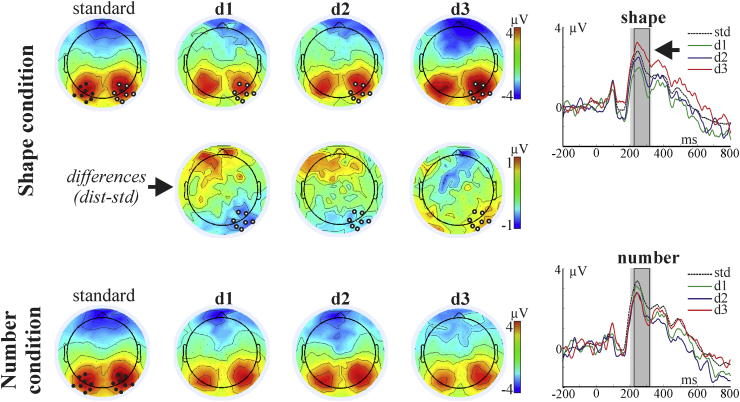
Left: Topographic plots of the P2 component from the significant time interval (220–320 ms) for the standard and for the three distance conditions. Difference (distance minus standard) topographic plots are presented for the Shape condition. The 16 electrodes entered into the analysis are marked on the standard plot. Electrodes which showed the significant distance effect (after FDR correction, see main text) are marked on the distance plots. Right: Time domain ERP, averaged from the electrodes showing the significant distance effect in the Shape condition. The significant (FDR corrected) time interval is marked with grey. The time interval initially entering the analysis is marked by light grey.

**Fig. 4:**
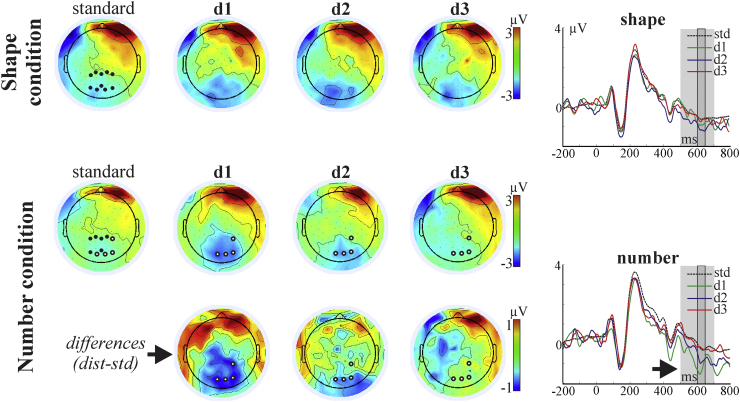
Left: Topographic plots of the late posterior component from the significant time interval (600–640 ms) for the standard and for the three distance conditions. Difference (distance minus standard) topographic plots are presented for the Number condition. The 10 electrodes entered into the analysis are marked on the standard plot. Electrodes which showed the significant distance effect (after FDR correction, see main text) are marked on the distance plots. Right: Time domain ERP, averaged from the electrodes showing the significant distance effect in the Number condition. The significant (FDR corrected) time interval is marked with grey. The time interval initially entering the analysis is marked by light grey.

ERP components evoked by the stimuli are illustrated in Fig. 2, Fig. 3, Fig. 4. The main early visual ERP components (P1 and N1), P2p, and the late positive component were identified based on the topography, polarity and latency parameters. P1 (around 100 ms post-stimulus), N1 (approximately 100–200 ms post-stimulus), and P2p (approximately 200–300 ms post-stimulus) components are typically recorded over the posterior region of the scalp (e.g. Dehaene, 1997). The late positive component is characterized by a later onset (after 300 ms) and with a centro-parietal distribution (Friedman & Johnson, 2000). Difference topographic plots for these components, by subtracting each distance condition from the standard, were also created (see Fig. 2, Fig. 3, Fig. 4). Based on the topographic distribution of the ERP components, and also on the distance-standard contrasts indicating distance-related processing, spatial regions with a contained and consistent pattern of electrodes were selected for further statistical analyses. For the identification of these spatial regions of interest, a similar approach to the one introduced by Dehaene (1996) was used. Point-by-point ANOVAs with the factor of distance within each oddball condition were carried out and electrodes with more than 10 consecutive datapoints (20 ms) showing the significant effect (*p* < 0.05) were included in further analyses. In the next step, instead of selecting one or two electrodes from the significant set, analysis was carried out on the whole set and false discovery rate was also controlled.

Since the point-by-point ANOVA involves several statistical tests, corrections of the individual tests’ thresholds is necessary in order to avoid the inflation of false positives. The matrices of *p*-values obtained from the point-by-point ANOVAs were corrected for false discovery rate (FDR) utilizing the method described by Benjamini and Yekutieli (2001). The FDR correction has been shown to be an effective practice for neuroimaging data where multiple-testing across related spatial and temporal datapoints is a common problem (Benjamini & Yekutieli, 2001), and where more conservative methods, like the Bonferroni correction controlling for the type I error rate, do not offer a good solution (Genovese, Lazar, & Nichols, 2002). Results were deemed significant when the false discovery rate among the rejected tests was estimated to be lower than 5%.

For the P1 and N1 components, 50 time points between 20 and 120 ms, and between 100 and 200 ms post-stimulus from 16 neighbouring electrodes from the temporal-parietal (as shown in Fig. 2) were submitted to point-by-point ANOVAs testing for the within-subject distance effect, separately for the Number and Shape conditions. The same approach was applied on the P2p component: data from points between 200 and 320 ms from 16 electrodes (see Fig. 3) were submitted to the point-by-point ANOVA.^2^ For the late posterior component, time points from between 500 and 700 ms interval from 10 temporal electrodes were submitted to the point-by-point ANOVAs. Summary *F*-values for the significant, FDR corrected point-by-point ANOVAs are reported. The Greenhouse–Geisser correction has been applied and reported where appropriate to correct for the violations of the sphericity assumption.

Supplementary material related to this article can be found, in the online version, at http://dx.doi.org/10.1016/j.biopsycho.2014.09.006.

**Supplementary Fig. 1:**
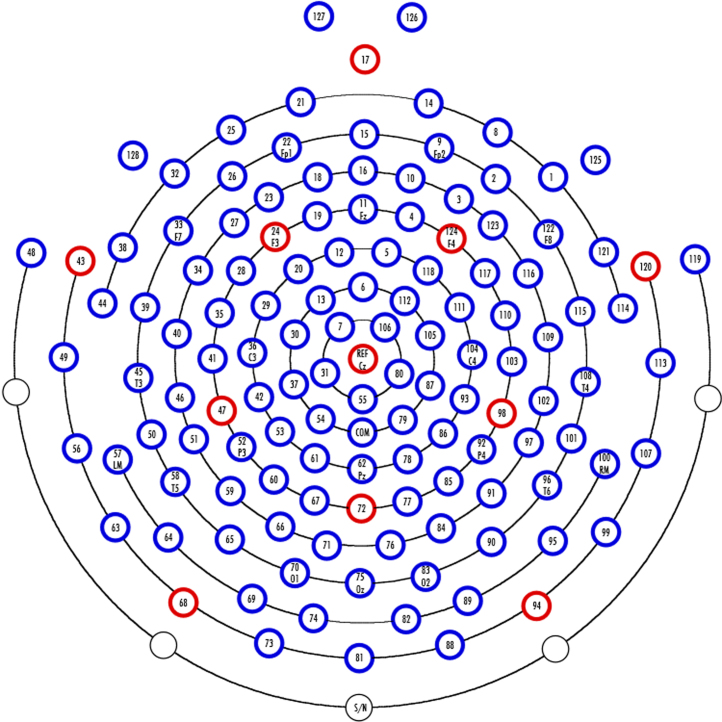
Electrode layout (128 electrodes, EGI Geodesic sensor net).

In the figures, both the tested (with FDR correction) and the significant time intervals, with the corresponding electrode groups (both the tested and the significant groups), are marked. Mean amplitude values from the electrode subgroup and within the significant time interval^3^ were then submitted to two-way ANOVAs to test for the modulations of distance effect by the oddball condition (Shape or Number). Mean amplitude values (instead of peak amplitudes) also prevent possible biases arising from the difference between trial numbers in the standard and in the deviant condition, meanwhile keeping statistical power (Luck, 2014). Post hoc tests (correcting for multiple comparisons with the Tukey-Kramer method) were performed in order to investigate the source of the significant Condition × Distance interactions.

## 3 Results

### 3.1 Behavioural and subjective responses

Target detection rate (for the cover task) was 100%. According to the short questionnaire, seventeen volunteers, out of 19, believed that there was no system in the changes in the display and deemed it random. One volunteer thought that size and number were related in some way, and that fewer items were perhaps larger. Another volunteer thought that there were hidden patterns (e.g. animals) in the displays. These responses confirm that our volunteers did not explicitely find any systematic relationships between number and size in this experiment.

### 3.2 ERP responses

**Fig. 5:**
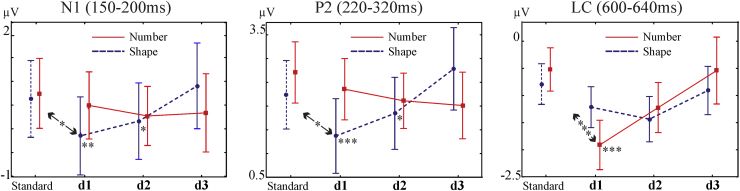
Representation of the two-way ANOVAs (Condition × Distance) for the three ERP components. ***p < 0.001; **p < 0.01; *p < 0.05. Mean and standard error (in ηV) values are shown in supplementary Table 1.

The results are summarized in Fig. 5. There was a significant interaction of Condition (Shape or Number) and Distance (standard, distance 1, d2, and d3) in the amplitude of both the N1 and P2^4^ ERP components; distance exerted a significant (and linear) effect in the Shape condition, but there was no effect of numerical distance in the Number condition. In the Number condition, distance effect was significant in the amplitude of the late posterior ERP component, ∼600 ms after stimuli presentation. Furthermore, in order to make sure that differences between the conditions are not due to the possibly larger variability in the Number deviant trials compared to the Shape deviant trials, the Bartlett's test for equal variance was performed for each deviant condition. Even though number deviant conditions constituted of two different trials (both smaller, and larger than the standard stimuli), the variances between Shape and Number deviant conditions were not significantly different from each other (N1: d1: *χ*^2^ = 0.48, *p* = 0.49; d2: *χ*^2^ = 0.48, *p* = 0.26; d3: *χ*^2^ = 2.53, *p* = 0.11; P2: d1: *χ*^2^ = 1.74, *p* = 0.19; d2: *χ*^2^ = 1, *p* = 0.31; d2: *χ*^2^ = 2.05, *p* = 0.15; LC: d1: *χ*^2^ = 1.34, *p* = 0.25; d2: *χ*^2^ = 0.63, *p* = 0.43; d3: *χ*^2^ = 3.54, *p* = 0.06). However, please note that the variability of the late component in the Number d3 condition was marginally more variable than the variability in the Shape d3 condition. But importantly, neither P1 nor N2 showed larger variability in the Number condition (all *p*s > 0.1), hence the lack of distance effects in the Number condition cannot be explained by the large variability. Variance is shown in Fig. 5 (error bars). None of the ERP responses differed between Shape and Number in the standard condition (all *p* > 0.66).

Supplementary Table 1 related to this article can be found, in the online version, at http://dx.doi.org/10.1016/j.biopsycho.2014.09.006.

Supplementary Table 1 Mean and standard error (in ηV) of the three ERP components, for each condition. Please also see Fig. 5.

There were no significant effects in the early visual P1 ERP component; it is omitted from the further reporting.

#### 3.2.1 N1 ERP component

Fig. 2 illustrates the findings. According to the point-by-point statistics, significant effects of distance emerged between 150 and 190 ms, at a group of 6 neighbouring right occipito-temporal electrodes, in the Shape condition (800 tests, of which 85 were significant after FDR) (average *F*(3,48) = 6.37, *ɛ* = 0.72, *p* = 0.004, *η*^2^ = 0.28). There were no significant effects of numerical distance in the N1 component in the Number condition, across all the tested electrodes and time points (see Fig. 2; all *p* > 0.84, *η*^2^ = 0.05). Most importantly, a two-way ANOVA with Condition (Shape, Number) and Distance (4 levels) resulted in a significant Condition × Distance interaction (*F*(3,48) = 3.38, *ɛ* = 0.83, adj. *p* < 0.035, *η*^2^ = 0.17). Post hoc comparisons indicated a linear effect of distance among the three Shape distance conditions: d3 significantly differs from both d1 and d2 (*p* < 0.002 and *p* < 0.035, respectively, and d1 differs from the standard: *p* < 0.05; see also Fig. 5). Post hoc comparisons showed that none of the distances differed from one another in the Number condition (all post hoc comparisons *p* > 0.99; if uncorrected for multiple testing: *p* > 0.41).

#### 3.2.2 P2(p) ERP component

Results are shown in Fig. 3. The effect of distance was significant in the Shape condition, between 220 and 320 ms, over 7 neighbouring left occipito-parietal electrodes (960 tests, of which 220 were significant after FDR) (average *F*(3,48) = 8.57, *ɛ* = 0.7, adj. *p* < 0.0009, *η*^2^ = 0.35). There were no significant effects of numerical distance in the amplitude of the P2 component in the Number condition (all *p* > 0.48, *η*^2^ = 0.1). Again, the two-way ANOVA yielded a significant interaction of Condition and Distance (*F*(3,48) = 4.35, *ɛ* = 0.55, *p* < 0.03, *η*^2^ = 0.21). Post hoc comparisons reflected a stepwise effect of distance in the Shape condition, with distance 3 being significantly different from d1 and d2 (*p* < 0.02 and 0.0003, and d1 differs from the standard: *p* < 0.05; see also Fig. 5). The amplitude of the P2 component was unaffected by changes in numerical distance in the Number condition (all post hoc comparisons for the Number condition: *p* > 0.97; if uncorrected for multiple testing: *p* > 0.33).

#### 3.2.3 Late posterior component

There was a significant effect of distance in the Number condition, between 600 and 640 ms, at four neighbouring centro-parietal electrodes (1000 tests, of which 71 were significant after FDR) (average *F*(3,48) = 8.87, *ɛ* = 0.75, adj. *p* < 0.0004; *η*^2^ = 0.36). Results are shown in Fig. 4. There was no significant effect of distance in the Shape condition (all corrected *p* > 0.9). However, although the distance effect was significant in the Number condition and was not significant in the Shape condition, the interaction of Condition and Distance was not significant this time (*F*(3,48) = 2.1, *ɛ* = 0.75, adj. *p* = 0.13, *η*^2^ = 0.1).

## 4 Discussion

Utilizing the high temporal resolution of EEG in a passive oddball (adaptation) paradigm, we have compared the time course of change detection in numbers and shapes. Using a non-symbolic numerical paradigm we attempted to further optimize perceptual controls than in previous non-symbolic adaptation studies (Ansari et al., 2006, Cantlon et al., 2006, Piazza et al., 2004, Piazza et al., 2007). Most importantly, we have varied circumference both at the items’ (intensive) and at the summary of items (extensive) level, which has not been done previously. Although it is impossible to account for all the (visual) perceptual covariates of numerosity since these visual properties are interdependent, we have still introduced a larger level of inter-trial variability in visual cues across the trials making it difficult for the perceptual system to automatically, and quickly, detect or associate these variables with changes in numerosity.

We also have exploited the high density electrode net and explored the whole topography, instead of pre-selecting a couple of canonical electrodes. We applied restrictions (temporal and spatial consistency and then FDR), in order to handle issues arising from multiple testing. The procedures used are relatively conservative (compared to the ERP literature), and similar methodologies have been suggested elsewhere (e.g. Maris & Oostenveld, 2007). There is also no biological reason to expect and measure experimental effects on only a couple of electrodes, especially since modern day computational power and technical development allow for more. We believe that the chance of finding random effects and also that of missing real effects is higher when only a couple of pre-defined electrodes are being looked at. Furthermore, using high density nets many electrodes will pick up voltage change from a single generator in the brain; so it is actually necessary to analyze the whole topography.

In summary, we have found significant, parametric changes in the early ERP components (N1 and P2) in function of changes in shape while these ERP components were insensitive to changes in number. These findings support our initial hypothesis, namely that there is no indication of early and automatic magnitude processing (P2p) when there is no consistent perceptual variable reliably co-varying with numerosity.

The missing distance effects in the Number condition are not merely null findings; the numerical distance did not exert any effects on the P1 and N2 ERP components, *in contrast* to the significant effects in the Shape condition. Furthermore, these differences between the shape and Number condition were not due to differences between the variance of the two conditions. The effects of numerical distance arose much later, approximately 600 ms after stimuli presentation.

In detail, effects of shape change have been found at two separate time windows. The first effect was detected after the P1 peak, between 150 and 190 ms. The occipito-parietal positivity change is reminiscent of the change-related positivity (CRP) usually detected in visual matching paradigms where visual attributes of the stimuli change between pairs of sequentially presented stimuli (Kimura et al., 2005, Wang et al., 2003, Fonteneau and Davidoff, 2007). The second effect of shape change emerged around and after the N2 ERP peak, between 220 and 320 ms. Changes in this deflection have previously been identified as the N270 ERP component. The N270 reflects the detection of conflict between a representation built up from previous stimuli and between the current stimulus. The N270 has been found to be modality-nonspecific and has been linked to the family of the mismatch-related components, including mismatch negativity (MMN; Näätänen, Gaillard, & Mäntysalo, 1978), error-related negativity (ERN; Gehring, Coles, Meyer, & Donchin, 1990), and N400 (Kutas & Hillyard, 1980). The N270 can be evoked by several different types of stimulus features, such as colour (e.g. Tian, Wang, Wang, & Cui, 2001), position (Yang & Wang, 2002), and more relevantly shape (Wang et al., 2004, Wang et al., 2003) and digit value (Kong et al., 2000). Both the CRP and the N270 are sensitive to unattended changes in stimuli features, reflecting an automatic, fast and unconscious system detecting changes in the environment. This is in accord with the notion that shape is a basic feature of visual stimuli which is unintentionally monitored by the perceptual system and changes are detected in a fast and automatic manner.

Interestingly, the largest difference emerged between the standard and the d1 stimuli, in both cases (and also in the Number condition). At the present moment, we cannot provide sufficient explanation for this finding. One would expect that the larger the difference from the standard, the larger the effect in the amplitude of the ERPs. Contrary to this expectation, the results rather indicate that more similar stimuli (as compared to the standard) evoke larger ‘efforts’ of perceptual discrimination however, without further investigation, our explanation should be regarded as speculation.

Regarding the Number condition, a significant interaction between Condition (Shape or Number) and Distance (4 levels) indicated that changes in number have not elicited changes in the amplitude of the N1 and P2 ERP components. The significant interaction between Shape and Number conditions in the early ERP components, and the significant distance effect in the late posterior component in the Number condition both indicate that the experimental paradigm and methodology were sensitive to the electrophysiological markers it intended to measure. Furthermore, the numerical distance effect in these early components was absent without any statistical indication towards statistically weak or ‘trendlike’ effects. None of the tested points showed close to significant distance effect in the Number condition from the time interval of the early ERP components (all *p* > 0.48); and even the uncorrected post hoc comparisons did not indicate trends that would suggest any differences among the three distance levels (all *p* > 0.33).

These results are in conflict with previous findings. Earlier ERP studies of numerical cognition have detected numerical distance effects earlier, already at around 180–200 ms after stimulus presentation. These early distance effects were taken as an indication that the representation of numerical values is activated within 200 ms, irrespective of whether the numerical magnitude represented was relevant to the task (Dehaene, 1996, Pinel et al., 2001, Soltész et al., 2007, Szűcs and Soltész, 2008, Temple and Posner, 1998). If the representation of numerical magnitudes is domain-specific and accessed automatically similar to other perceptual features, as theoretised (Piazza et al., 2004), then what might be the source of discrepancy between the results of prior studies and our current findings?

One possible explanation for the lack of these early numerical distance effects in the present case might lie in significant methodological differences between these studies. First of all, here we employed a non-symbolic paradigm where numerical magnitude was irrelevant, hence unattended by the volunteers. Second, and most importantly, we have introduced more stringent perceptual controls by carefully varying for the items’ individual and as well as summated circumference (see Section 2). Items’ intensive and extensive circumference has not been taken into account in previous non-symbolic adaptation studies (Cantlon et al., 2006, Piazza et al., 2004, Piazza et al., 2007), leaving a systematic and possibly significant confound in these previous designs. Co-varying circumference could well provide a perceptual cue for numerosity changes; in fact, 6–8 months old children detected change in circumference but not in number, when these two properties were contrasted with each other (Clearfield & Mix, 1999).

In line with some previous arguments (Gebuis and Reynvoet, 2011, Gebuis and Reynvoet, 2012a, Gebuis and Reynvoet, 2012b, Gebuis and van der Smagt, 2011) we suggest that there is an ecologically reasonable explanation as to why there might not be a hardwired representation of numerical magnitudes per se (e.g. in the IPS. See also Shuman and Kanwisher (2004) for a non-specific account of the IPS). Among natural circumstances, the numerical magnitude of objects usually correlate almost perfectly with several simple perceptual cues. Usually, ten apples occupy a larger space than six apples. Without enumerating apples, when we want more of them, we can be equally successful in our choices if we merely rely on for example visual surface area instead of abstract concept of numbers. From an evolutionary perspective, if survival is secured by processing these perceptual cues, why should we expect a perceptual system separately specialized to abstract numerical magnitudes? According to this train of thought, number per se is a higher-level property assembled from lower-level sensory properties by our perceptual system. The perception of numbers may only reach an abstract level, independent of co-varying perceptual variables, either when computation is explicit or when abstract (i.e. free from natural perceptual co-variates) symbols are used. In both these latter cases, it is required that the abstraction is explicitly understood or already learned by the subject. The relatively late appearance of the ERP numerical distance effect in the present study could well reflect that the numerical feature of the displays is either assembled from the earlier registered continuous physical variables, or detected only later as a by-product of the fewer, but still correlating physical properties. The later extraction of number can happen because even if controlling for most of the perceptual co-variates across trials, there are still some co-variates per each individual trial providing a helpful cue for the perceptual system. For example, if individual items’ surface is controlled for in one display, the summary of the items’ surface would still correlate with number and vica versa. Even if both the intensive and extensive variables are drawn from a certain distribution to avoid a perfect linear correlation between number and these perceptual parameters, individual displays with deviant numerosities would inevitably fall to either of the extreme ends of the distribution. Thus, numerically deviant displays are inevitably deviants in comparison to the average of the distribution of perceptual values.

## 5 Conclusion

Corroborating our earlier behavioural findings (Soltész et al., 2010, Szűcs et al., 2013), our current results suggest that non-symbolic numerosity is not automatically detected without correlating visual cues unless attention is drawn to the concept of abstract numbers. We have found early EEG signatures of change detection/violation of expectation for changes in shape. Meanwhile, the registration of changes in numerical magnitude occured later. Numerical distance effects have not been found in the time interval suggested by earlier studies and by the theory of an abstract, automatic hard-wired representation of approximate numerical magnitudes. We suggest that under more strictly controlled circumstances (i.e. variation of perceptual cues), the abstract concept of numerosity is constructed at a later stage of cognitive processing. We also conclude that abstract number is not a basic property of visual stimuli, but rather is derived from inevitably co-varying perceptual features possibly after automatic grouping (gestalt principles) and categorization processes.

## Limitations

First, as usual in neuro-imaging studies we have chosen to correct for the inflated false discovery rate (Benjamini & Yekutieli, 2001), instead of correcting for the false positive rate. FDR has been reported to be suitable for imaging data and we believe that the pattern of the results is consistent and reliable. However, it should be noted that the false positive rate (in the present case mostly in the Shape condition) remains unknown.

Second, we have defined numerical distance conditions by including both smaller and larger magnidudes (e.g. 6 and 24 as d3). This grouping method did not allow us to see whether perception of smaller and larger magnitudes were different from each other. But importantly, the lack of distance effects in the Number condition cannot be due to possible larger variances in the numerical distances, than in the shape distances. Third, although there were no magnitudes from the subitizing range (Trick & Pylysyn, 1994), there were numbers still smaller than 10 in the current paradigm. Numbers below or around 10 might also be processed differently (i.e. ‘embodied’) than larger magnitudes (Domahs, Moeller, Huber, Willmes, & Nuerk, 2010).

Third, we have classified our findings in the second time-interval as an N270 ERP component. Similar effects in a similar time range could also be identified as a P2 ERP component, sensitive to categorical changes in shape (Koester & Prinz, 2007). Here we do not have sufficient evidence to be able to functionally discriminate between these components.

## Conflict of interest

The authors declare that they have no conflict of interest.

## Footnotes

1 Overall surface: *N* × 2*πr*, while overall circumference is: *N* × *πr*^2^.

2 Electrode labels for N1 and P2: 51, 58, 59, 63, 64, 68, 69, 70, 83, 89, 91, 94, 95, 96, 97, 99. For the late component (10 electrodes): 60, 61, 62, 69, 74, 75, 78, 82, 85, 89. All electrode locations are shown in Supplementary Fig. 1.

3 Selecting time intervals based on point-by-point statistics, and then averaging time points from the significant time interval, is not without example in the ERP literature (see for example: Dehaene (1996)). In addition, in the present study we also made an attempt to control for the several tests yielded by the point-by-point analysis.

4 From here onwards P2 and not P2p, because P2p refers to the number-specific component.
