# Supplementary material for: Neural adaptation to non-symbolic number and visual shape: An electrophysiological study
Source: Biol Psychol. 2014 Dec;103:203–11. doi: 10.1016/j.biopsycho.2014.09.006 (PMC4266538; doi:10.1016/j.biopsycho.2014.09.006)
Supplement: Supplementary Table 1 — Mean and standard error (in ηV) of the three ERP components, for each condition. Please also see Fig. 5. [file mmc1.docx]

|  |  | **N1 component** | **P2 component** | **Late posterior component** |
| --- | --- | --- | --- | --- |
| **Shape condition** | **Standard** | 0.63 (0.8) | 2.24 (0.72) | -0.79 (0.37) |
|  | **D1** | 0.15 (0.82) | 1.37 (0.79) | -1.21 (0.38) |
|  | **D2** | 0.16 (0.8) | 1.85 (0.76) | -1.44 (0.41) |
|  | **D3** | 0.9 (0.9) | 2.79 (0.87) | -0.91 (0.45) |
| **Number condition** | **Standard** | 0.75 (0.74) | 2.7 (0.65) | -0.52 (0.4) |
|  | **D1** | 0.5 (0.7) | 2.35 (0.64) | -1.91 (0.46) |
|  | **D2** | 0.27 (0.62) | 2.1 (0.68) | -1.22 (0.46) |
|  | **D3** | 0.34 (0.82) | 2 (0.7) | -0.53 (0.62) |

Supplementary table 1
